# Modeling *TCIRG1* Neutropenia by Utilizing Patient Derived Induced Pluripotent Stem Cells

**DOI:** 10.33696/immunology.7.228

**Published:** 2025

**Authors:** Vahagn Makaryan, Merideth L. Kelley, Audrey Anna Bolyard, Chris Cavanaugh, Jennifer Hesson, Julie Mathieu, Michael J. Lenaeus, David C. Dale

**Affiliations:** 1 Department of Medicine, University of Washington, Seattle, Washington, U.S.A.; 2 Ellison Stem Cell Core, Institute for Stem Cell and Regenerative Medicine, UW Medicine at SLU, Seattle, Washington, U.S.A.; 3 Department of Comparative Medicine, University of Washington, Seattle, Washington, U.S.A.

**Keywords:** *TCIRG1*, Neutropenia, Granulopoiesis, Neutrophils, iPSCs, CRISPR/Cas9, V-ATPase

## Abstract

Congenital neutropenia is characterized by a reduced neutrophil count, decreased innate immunity and increased susceptibility to recurrent infections. While congenital neutropenia has various genetic causes, recent studies have linked *TCIRG1* mutations to this condition. *TCIRG1*, a key component of the vacuolar ATPase (V-ATPase) complex, is essential for osteoclast function, but its role in hematopoiesis remains unclear. We previously identified heterozygous *TCIRG1* mutations, including R736S, R736C, R736P, and E722D, in individuals with congenital neutropenia. However, the mechanism by which these mutations lead to impaired granulopoiesis remains unknown.

To investigate the functional consequences of *TCIRG1* mutations, we generated induced pluripotent stem cells (iPSCs) from affected individuals and healthy controls. Using *in vitro* differentiation protocols, we assessed hematopoietic progenitor formation, proliferation, survival, and neutrophil differentiation. We observed significant defects in myeloid differentiation and increased cell death in patient-derived iPSC lines. CRISPR/Cas9-mediated correction of the R736C mutation restored normal neutrophil differentiation, confirming its pathogenic role. Immunofluorescence analysis revealed reduced expression and altered intracellular localization of the *TCIRG1* protein, characterized by a more diffuse cytosolic distribution in the mutant cell lines.

Our findings suggest that *TCIRG1* mutations impair neutrophil development, likely through structural and functional disruption of the V-ATPase complex. This study provides new insights into the molecular basis of *TCIRG1*-associated neutropenia and highlights potential avenues for therapeutic intervention.

## Introduction

Neutropenia, characterized by an abnormally low neutrophil count, is a hematological disorder that increases susceptibility to recurrent infections [1,2]. While neutropenia has diverse genetic and acquired causes, recent studies have implicated mutations in the *TCIRG1* (T-cell immune regulator 1) gene in a subset of cases [3–5]. *TCIRG1* is best known for its role in osteoclast function and bone resorption, with loss-of-function mutations linked to autosomal recessive osteopetrosis (ARO) [6–10]. However, emerging evidence suggests that *TCIRG1* mutations can also impair neutrophil development and survival, leading to congenital neutropenia.

Despite its established role in bone homeostasis, the precise mechanism by which *TCIRG1* dysfunction results in neutropenia remains unclear. We previously identified a novel heterozygous missense mutation, R736S, in *TCIRG1*, as the cause of severe congenital neutropenia (SCN) in a large multigenerational family [3]. Additionally, we found a statistically significant correlation between lower absolute neutrophil counts (ANC) and rare *TCIRG1* missense variants in a population-based genome screening cohort [4]. More recently, we identified five other unrelated patients and families with congenital neutropenia harboring heterozygous E722D, R736C and R736P mutations. Independently, Shinwari *et al.* identified a novel V52L mutation in *TCIRG1*, also associated with a neutropenic phenotype [5].

The Genome Aggregation Database (gnomAD) contains extensive data on heterozygous *TCIRG1* variants in the general population, most of whom are healthy and exhibit no hematological abnormalities. However, why specific heterozygous *TCIRG1* mutations at R736 and E722 lead to neutropenia remains unknown.

In this study, we showed that repair of the R736C mutation with CRISPR/Cas9 editing of patient derived induced pluripotent stem cells (iPSCs) corrected the defect in myelopoiesis, and modeled *TCIRG1*-associated neutropenia using patient derived iPSCs, and characterized the hematopoietic abnormalities through *in vitro* differentiation studies.

## Materials and Methods

### Patients

Clinical information and biological materials for the study were obtained with informed consent in compliance with University of Washington regulations. All procedures were conducted in accordance with ethical standards and approved protocols to ensure the protection of participant rights and confidentiality.

In each family, the *TCIRG1* mutation shows perfect segregation, with every individual carrying the mutation exhibiting clinical manifestations consistent with the neutropenia phenotype.

**Figure 1.**
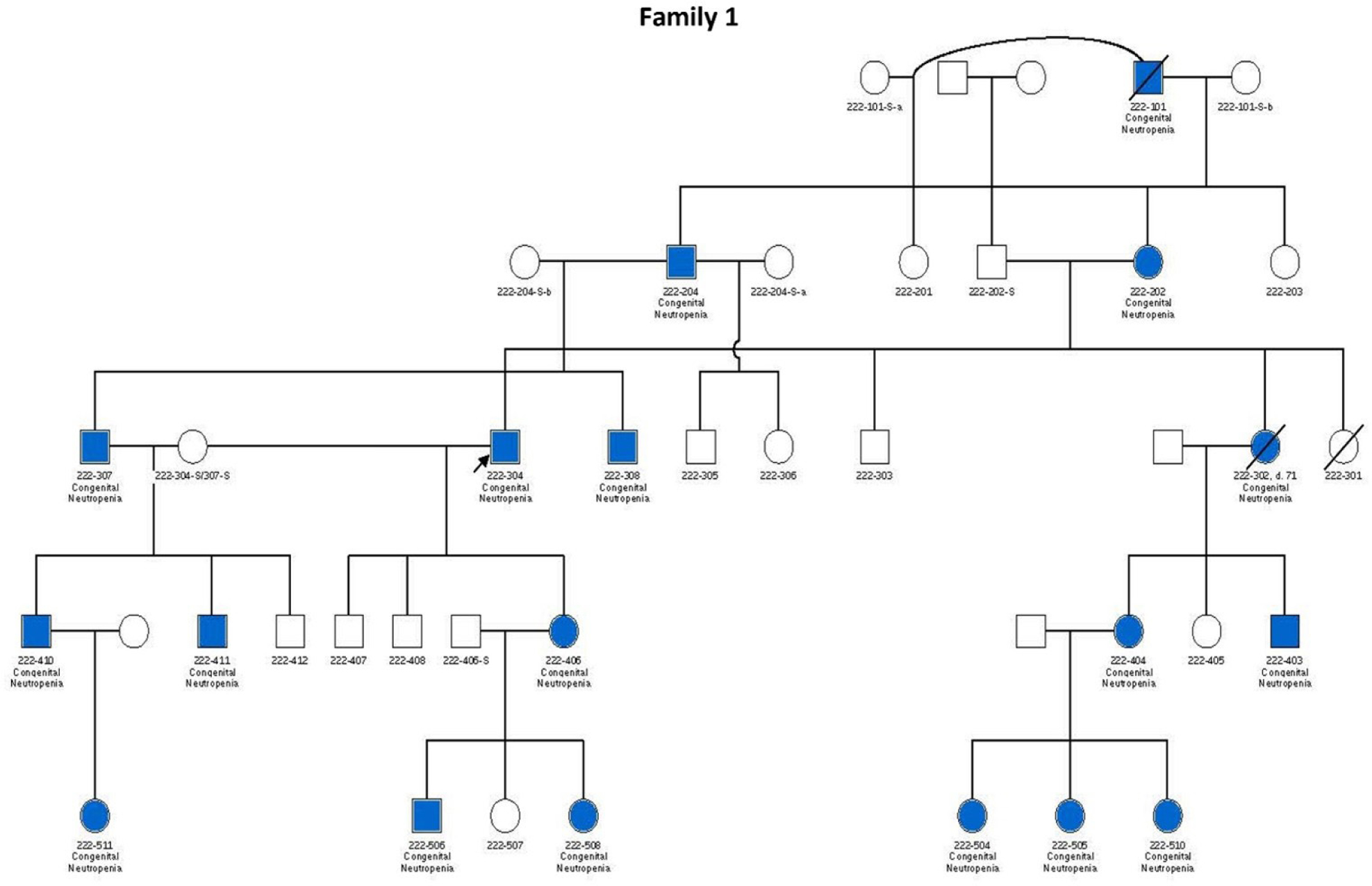

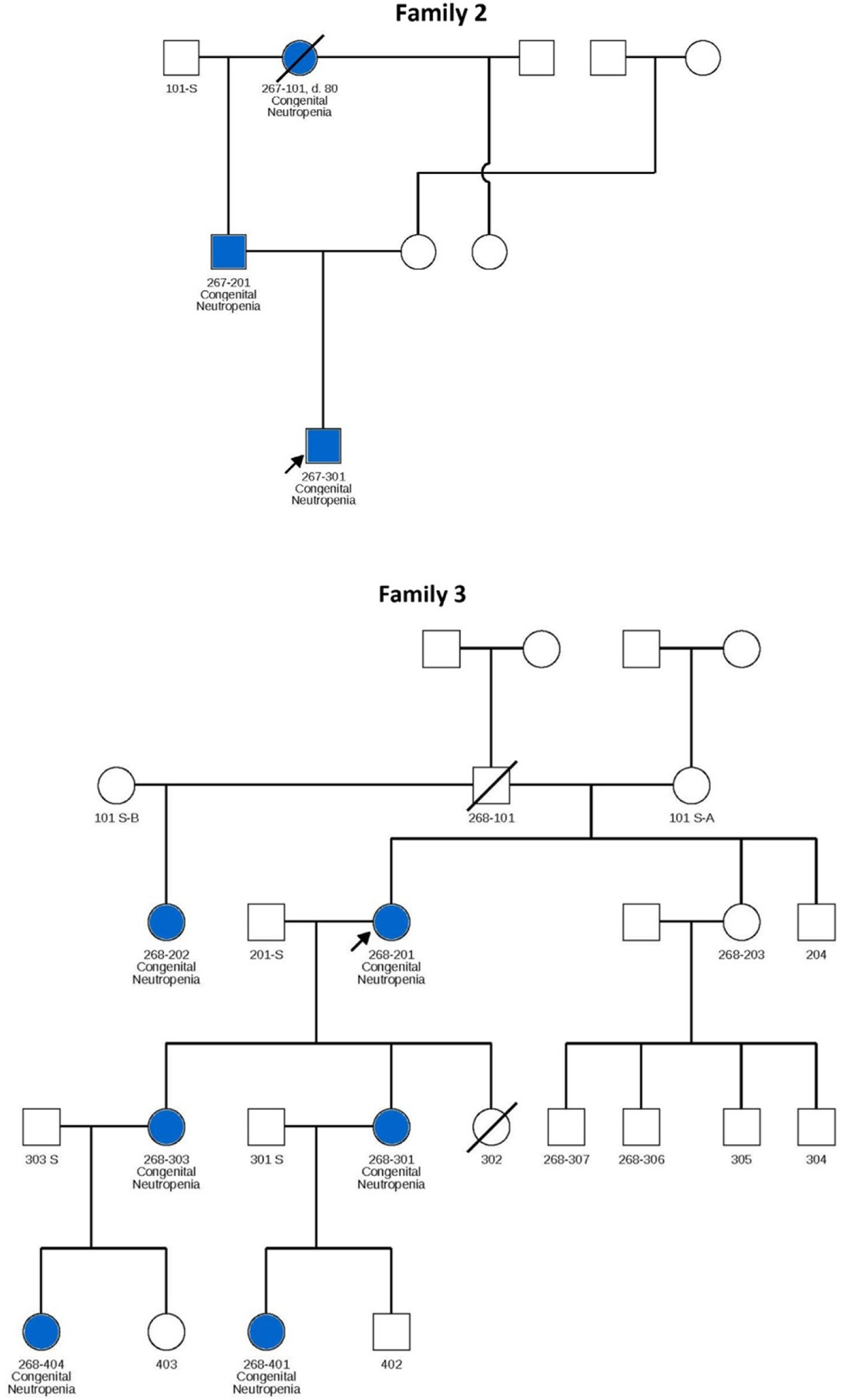

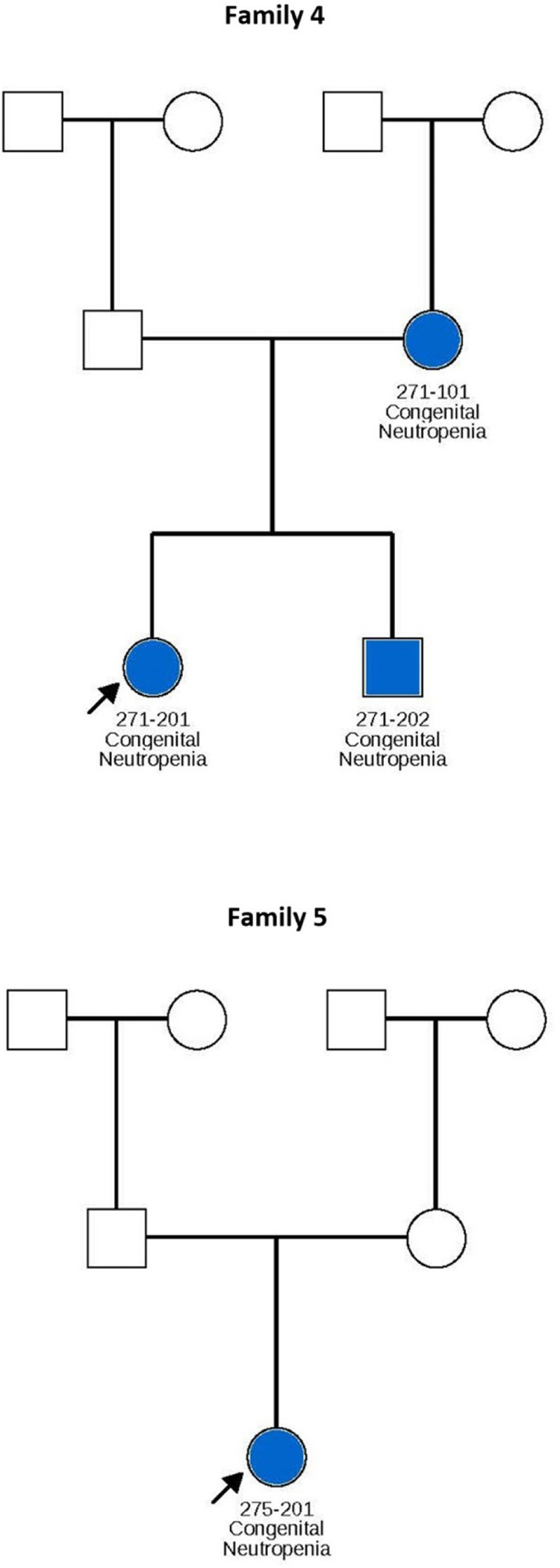

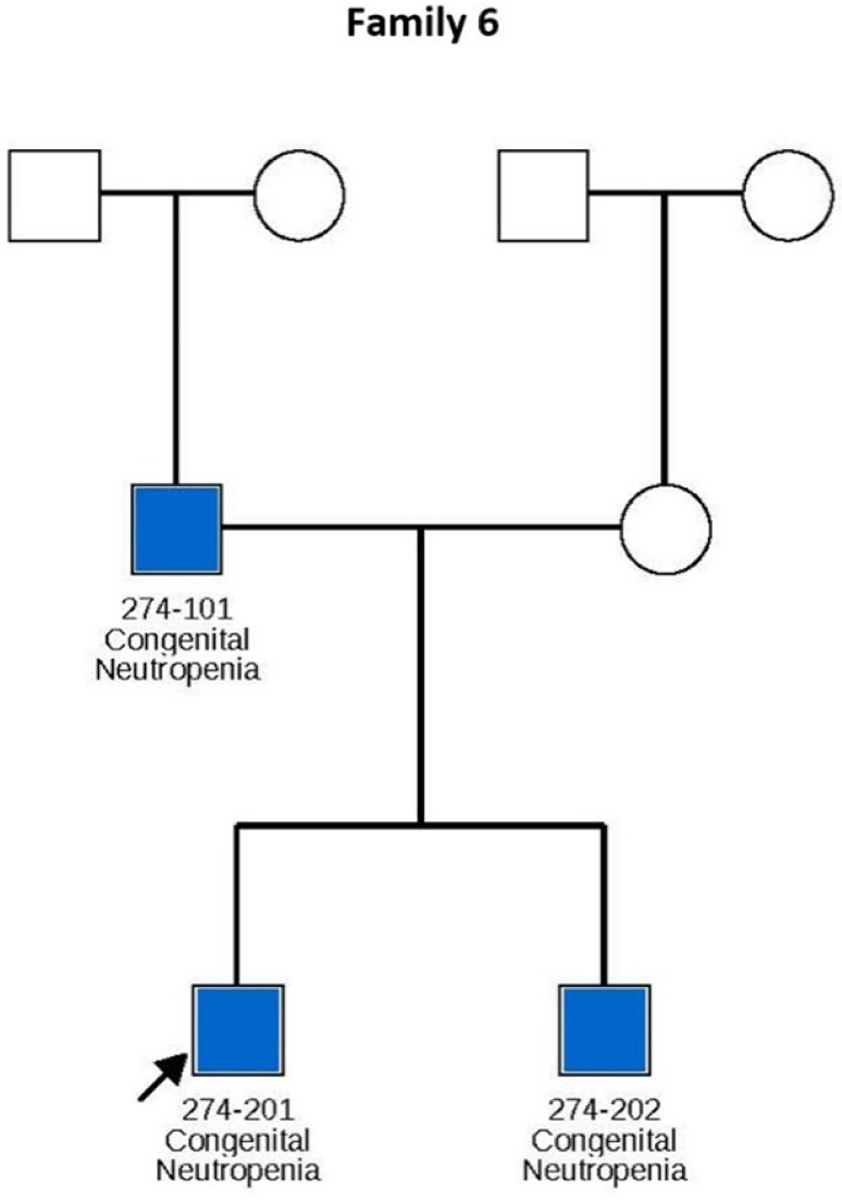
Pedigrees of six families carrying *TCIRG1* mutations, showing inheritance patterns and affected individuals. Subjects shown in blue are positive for the *TCIRG1* mutation and exhibit a neutropenia phenotype. Subjects shown in white are either negative for the *TCIRG1* mutation, not known to present any clinical symptoms, or lack available information on clinical presentation and mutational analysis. Arrows indicate the index patient in each family, through whom the rest of the family was identified and subsequently studied.

In this study, we analyzed cells derived from patients 307 and 411 from Family 1, hereafter referred to as patients 1 and 2, respectively, and 101 and 201 from Family 2 hereafter referred to as patients 3 and 4, respectively (see pedigrees in Figure 1). Patients 307 and 411 from Family 1 are severely neutropenic and have experienced recurrent severe infections, including mouth ulcers, skin infections, and perirectal and liver abscesses. Similarly, patients 201 and 301 from Family 2 exhibit severe neutropenia. Notably, Patient 301, a 5-year-old boy, has absolute neutrophil counts (ANCs) consistently below 0.2 × 10^9^/L even when appearing clinically well, but he remains highly susceptible to infections, including severe gingivitis and mouth ulcers. His father (Patient 201, age 53) also has persistent neutropenia with ANCs typically below 0.5 × 10^9^/L. Interestingly, Patient 101, despite carrying the same mutation, presents with milder neutropenia.

Patient 201 from Family 3 is severely neutropenic, with ANCs typically ranging between 0.2–0.3 × 10^9^/L. Her two daughters, patients 301 and 303, are also neutropenic, with ANCs usually below 0.5 × 10^9^/L, and suffer from recurrent mouth sores, sinusitis, and episodes of pneumonia. In Family 4, most affected members display relatively milder neutropenia, with ANCs ranging from 1.0 to 5.4 × 10^9^/L, though chronic mouth ulcers are common. Patient 201 from this family has more severe neutropenia and significant health complications (see Table 1).

Patient 201 from Family 5 carries the *TCIRG1* R736C mutation, which appears to have arisen *de novo*. She exhibits mouth ulcers, recurrent urinary tract infections, and mastitis complicated by abscess formation, with ANCs typically below 0.5 × 10^9^/L. Finally, affected members of Family 6 generally present with mild to moderate neutropenia, with ANCs ranging from 0.1 to 1.6 × 10^9^/L, and have experienced multiple hospitalizations due to pneumonia.

### iPSC reprogramming

Patient-derived skin fibroblasts (patients 1, 2, and 4) and whole blood (Patient 3) were collected with informed consent in accordance with institutional ethical guidelines (IRB# 00001762). Peripheral blood mononuclear cells (PBMCs) were isolated from whole blood using Ficoll density gradient centrifugation and cultured in complete PBMC medium (StemPro-34 SFM, Cat. no 10639011) supplemented with StemPro-34 Nutrient Suppliment (Thermo Fisher Scientific, Cat. no 10641) for 4–7 days. Both fibroblasts and PBMCs were reprogrammed into iPSC using CytoTune-iPS 2.0 Sendai Reprogramming Kit following the manufacturer’s recommendation (Thermo Fisher, Cat.no A16517). Cells were transduced at the recommended multiplicity of infection (MOI) per manufacturer’s guidelines and incubated at 37°C, 5% CO_2_ for 24 hours, after which the media was replaced and cells were maintained in expansion medium (StemPro + cytokines for PBMC and DMEM + 10% FBS for fibroblasts). On day 3 post-transfection, the PBMCs were transferred to irradiated mouse embryonic fibroblast (MEF) feeder layers and cultured in StemPro media without cytokines; the media was switched to DMEM/F12 medium supplemented with sodium butyrate (0.1mM) and SAHA (50nM) on day 7. On day 7 post-transduction, fibroblast cells were transferred to irradiated MEF in DMEM/F12 medium supplemented with sodium butyrate (0.1mM) and SAHA (50nM). Emerging iPSC colonies were identified between days 18–21, manually picked under a stereomicroscope, and transferred to fresh irradiated MEF plates. Once iPSC lines were established, they were adapted to feeder-free conditions on Matrigel-coated plates in mTeSR-Plus (STEMCELL Technologies, Cat. No 100–0276) and maintained for at least three passages with daily media changes and routine passaging using Accutase.

### CRISPR/Cas9 genome editing of iPSCs

CRISPR/Cas9 genome editing was used to generate the *TCIRG1* S733F mutation in iPSCs derived from a healthy donor (WTC-11, Coriell, #GM25256). The same approach was employed to correct the R736C mutation in patient-derived iPSCs (Patient 4). Cells were electroporated with a Cas9 ribonucleoprotein (RNP) complex, consisting of Cas9 (0.3 μM, Sigma, Cat.No CAS9PROT) and gRNA (1.5 μM, Synthego, CCGCCTCCTACCTGCGCCTG for S733F knock-in and CCGCCTCCTACCTGTGCCTG for R736C repair), along with a single-stranded DNA (ssDNA) donor template (2 μM, IDT) for homology-directed repair (HDR) (TCATGCACCAGGCCATCCACACCATCGAGTTCTGCCTGGGCTGCGTCTCCAACACCGCCTTCTACCTGCGCCTGTGGGCCCTGAGCCTGGCCCACGCCCGTGAGTGACCTGGCCACCGACG for S733F knock-in and TCATGCACCAGGCCATCCACACCATCGAGTTCTGCCTGGGCTGCGTCTCCAACACCGCCTCCTACCTGCGCCTGTGGGCCCTGAGCCTGGCCCACGCCCGTGAGTGACCTGGCCACCGACG for R736C repair). Electroporation was conducted using an Amaxa nucleofector (Human Stem Cell Kit 2) in the presence of a ROCK inhibitor to enhance cell survival and recovery post-transfection. To further optimize editing efficiency, cells were treated with HDR Enhancer v2 (IDT).

Following electroporation, cells were cultured in mTeSR Plus media (STEMCELL Technologies, Cat. No 100–0276). Individual iPSC colonies were manually hand-picked, expanded, and transferred into 96-well plates for further processing. Genomic DNA was extracted from each clone using QuickExtract (Epicentre, Cat. No QE09050), and a nested PCR strategy was employed to amplify the target region, ensuring high specificity and sensitivity for detecting edits. Purified PCR products were treated with EXO-SAP (ThermoFisher, Cat No 78205) to remove excess primers and nucleotides before being submitted for Sanger sequencing (Genewiz). Sequence analysis was then performed to confirm successful editing (Supplementary Figure 1).

### iPSC hematopoietic differentiation

iPSCs were differentiated into hematopoietic progenitors using STEMdiff Hematopoietic Kit (STEMCELL Technologies, Cat No 05310) following the manufacturer’s protocol. Briefly, iPSCs were maintained in feeder-free conditions before initiating differentiation. Colonies were dissociated into “embryonic body” aggregates and seeded in 12 well plates (STEMCELL Technologies, Cat No 200–0624) in hematopoietic differentiation medium, supplemented with provided cytokines and growth factors to promote hematopoietic lineage specification. On day 12 the floating cells were harvested and differentiation efficiency was assessed by flow cytometry for CD34 (Biolegend Cat.no 343510) and CD45 (Biolegend Cat.no 368508) expression.

### Myeloid differentiation

The resultant CD34^+^ cells were allowed to recover for 3 days in CD34^+^ expansion media and were subjected to a differentiation protocol as previously described [11,12]. In brief, HSCs were cultured for 7 days in RPMI (Cat.no 11875093, Gibco^™^) supplemented with 1% Glutamax (Cat.no 35050061, Gibco^™^), 10% FBS (Cat.no 04–001-1A, Biological Industries), 5 ng/ml IL-3 (Cat.no 200–03), SCF (Cat.no 300–07), GM-CSF (Cat.no 300–03) & 10 ng/ml G-CSF (Cat.no 300–23), all from PeproTech, for proliferation and myeloid progenitor differentiation followed by a 7-day culture in RPMI, 1% Glutamax, 10% FBS, 1% Penn Strep (Cat.no 03–031-1B, Biological Industries), 10 ng/ml G-CSF for neutrophil differentiation and maturation.

### Cytospin staining

8 × 10^4^ cells at day 14 of differentiation were spun onto Cytoslide microscope slides (ThermoScientific, Cat No 5991056) using Cytospin 4 low speed cytocentrifuge (Thermo Scientific) and stained with Kwik-Diff staining system (Shandon, Cat. No 9990700) according to manufacturer’s recommendations. Microphotographs were taken on LEITZ LABORLUX S polarizing light microscope at 400X magnification using Nikon DSLR digital camera.

### Flow cytometry

Myeloid maturation of CD34^+^ cells was analyzed at day 14 of the differentiation by flow cytometry utilizing antibodies characterizing the neutrophilic lineage. CD66b anti-human; Pacific Blue (Cat No. 305112, Biolegend), CD14 anti-human; APC (Cat No. 130–110-520), CD11b anti-human; APC (Cat No. 130–110-554) and CD15 anti-human; Pacific Blue (Cat No. 130–113-488) all from Miltenyi Biotec, unless indicated otherwise, were used. Cell debris was gated out by using a zombie yellow viability kit (Biolegend, 423103).

### Immunofluorescence analysis and TCIRG1 intracellular localization

Induced pluripotent stem cell (iPSC)-derived CD34^+^ hematopoietic progenitor cells were differentiated toward the myeloid lineage using a standard differentiation protocol as previously described [11,12]. Differentiation was carried out under optimized culture conditions for 14 days, with regular media changes to support myeloid lineage commitment.

At the end of the differentiation period, cells were harvested, spun onto slides and fixed with 4% paraformaldehyde for immunofluorescence staining. Cells were permeabilized with 0.2% Triton X-100, then blocked with 5% normal goat serum. Slides were incubated with rabbit anti-human TCIRG1 (1:100 dilution; Abcam, ab224654) and mouse anti-human myeloperoxidase (MPO) (1:20 dilution; R&D Systems, MAB3174) primary antibodies for 48–72 hours at 4°C in a humidified chamber, washed and then incubated for 40 minutes at room temperature with Alexa Fluor 647 conjugated polyclonal goat anti-rabbit IgG H&L (1:200 dilution; Abcam. ab150079) and Alexa Fluor 488 conjugated polyclonal goat anti-mouse IgG H&L (1:200 dilution; Abcam, ab150113) secondary antibodies. Cells were washed, then mounted using ProLong Gold with DAPI (Invitrogen, P36935) and visualized at the Keck Imaging Center (University of Washington) using a Leica DMI6000 widefield immunofluorescence microscope and 40x water immersion objective.

## Results

### iPSCs hematopoietic differentiation

To understand the specifics of the mutations in the positions R736 and E722, we have also created another mutant iPSC line harboring heterozygous S733F mutation utilizing CRISPR/Cas9 technology. We selected this particular variant due to its proximity to R736 site and its high prevalence in the healthy population. Individuals with heterozygous variant of S733F are healthy and do not have any hematological abnormalities. Homozygous S733F mutations are causing osteopetrosis. We are using this cell line along with 2 healthy volunteer lines for comparative studies along with cell lines from the affected families.

**Figure 2.**
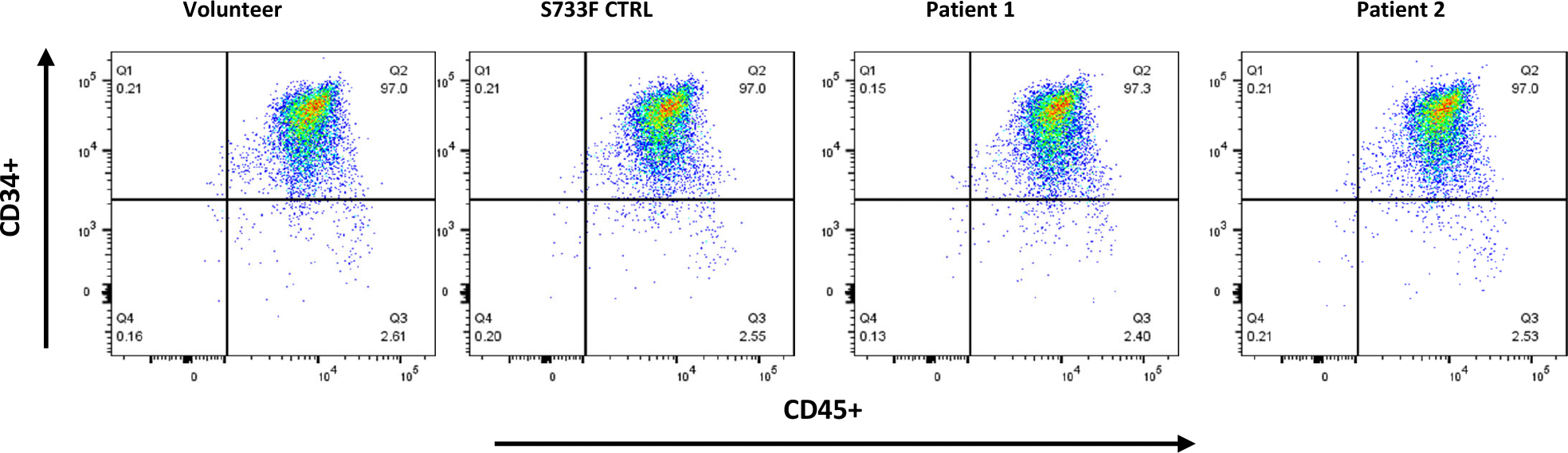
Hematopoietic differentiation of patient derived and control iPS cells. iPSCs derived from affected individuals from Family 1 and Family 2, as well as iPSC lines from a healthy volunteer and a line harboring the S733F mutation, were directed toward hematopoietic differentiation using the STEMdiff Hematopoietic Kit (STEMCELL Technologies). A representative flow cytometric analysis demonstrates the expression of CD34 and CD45 surface markers on the recovered cells following differentiation.

Four iPSC lines from the Family 1 and 2 harboring R736S and R736C mutations, as well as iPSC lines derived from a healthy volunteer and the line harboring S733F mutation were pushed towards the hematopoietic differentiation utilizing STEMdiff Hematopoietic Kit (STEMCELL Technologies). All lines generated comparable CD34^+^/CD45^+^ myeloid progenitor cell populations (Figure 2).

### Myeloid differentiation: proliferation, cell survival, and neutrophil formation

**Figure 3.**
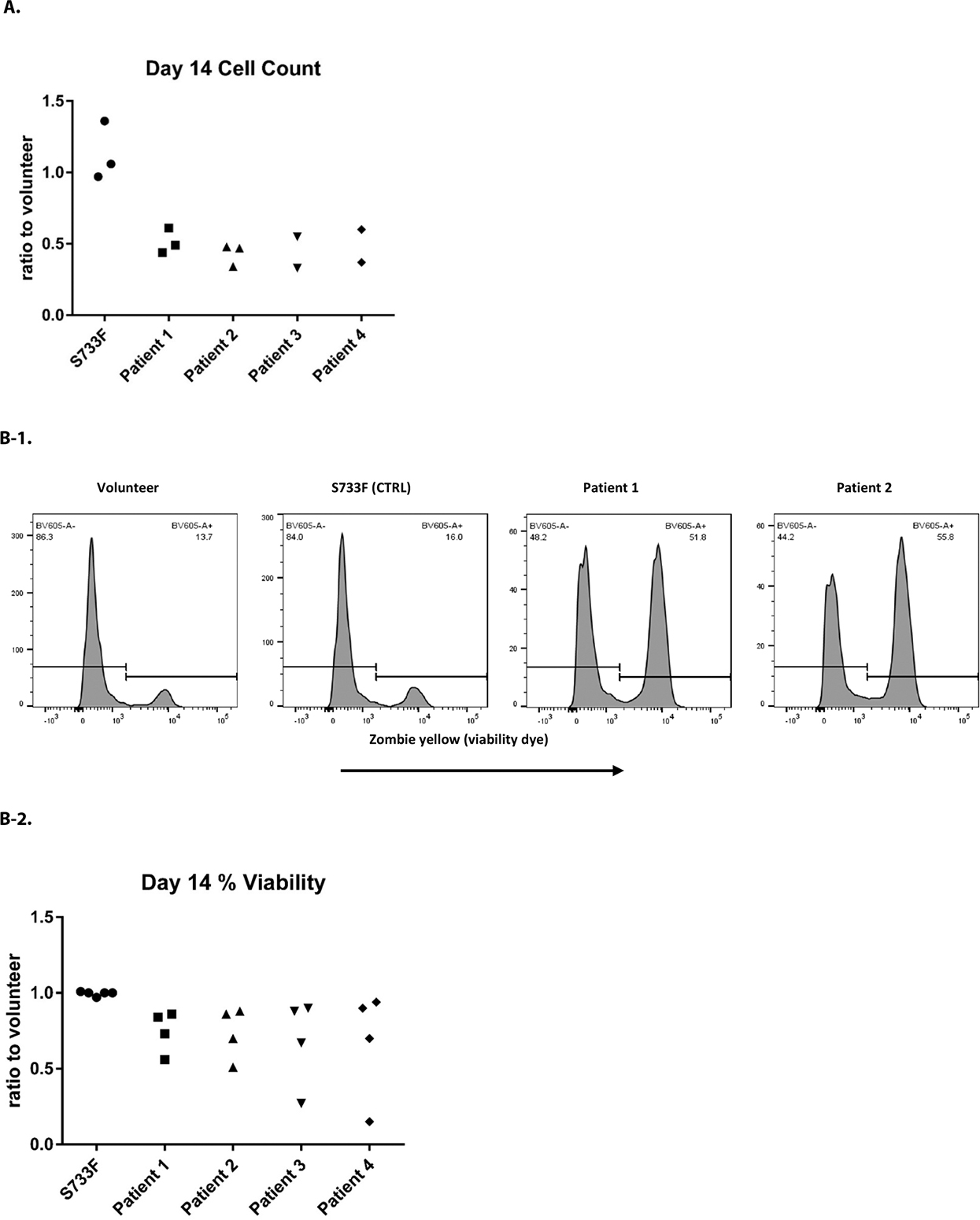

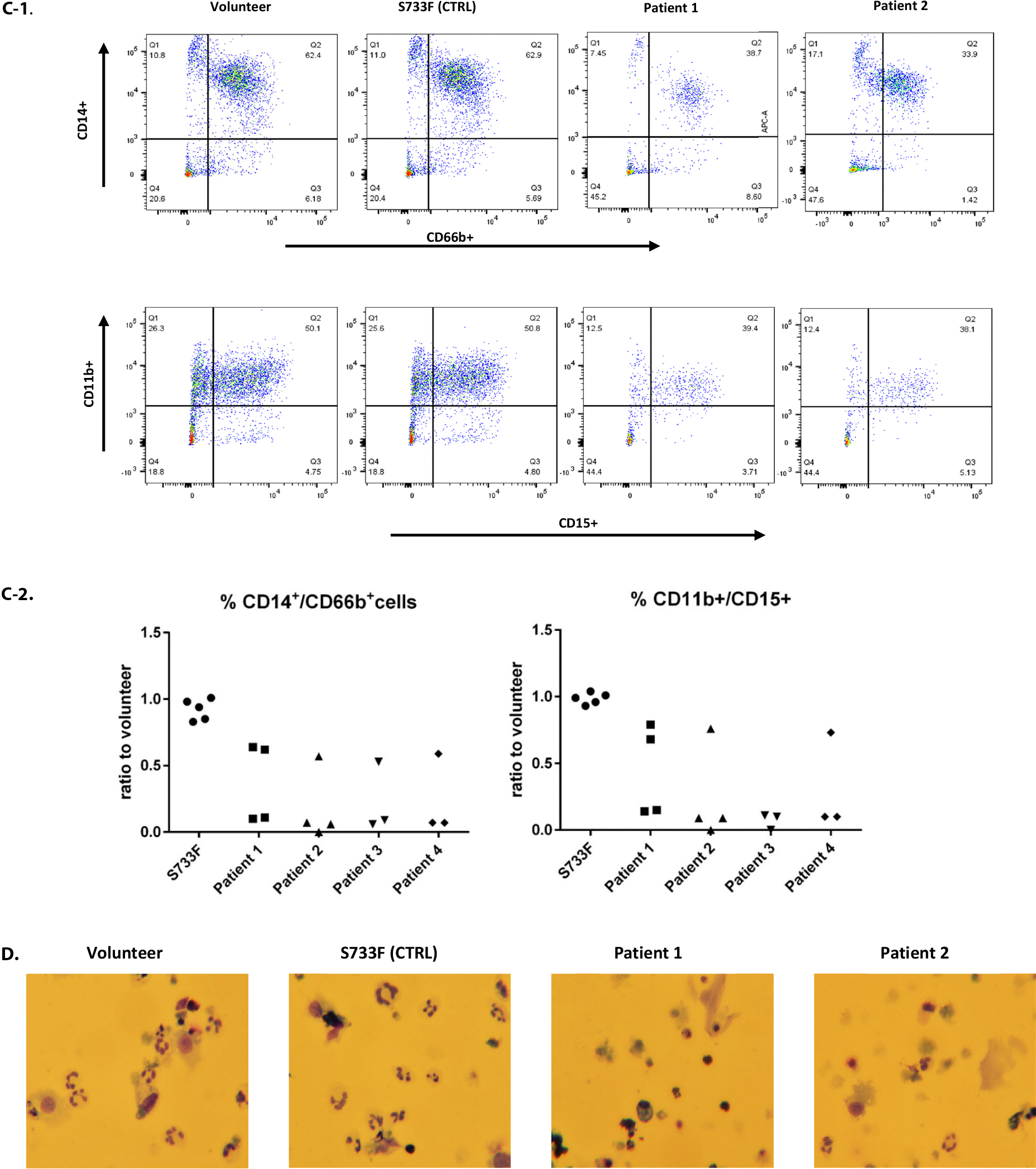
Proliferation, survival and differentiation of iPSC derived CD34+ hematopoietic cells. iPSC derived CD34+ cells pushed towards the myeloid differentiation using the standard protocol previously described [11,12]. **A.** Cell proliferation was evaluated at day 14 of differentiation by using Moxi V automated cell counter and the number of live cells recorded. For each individual experiment, the total number of patient cells was divided by the percentage measured from normal volunteer (control) cells, and this ratio was plotted. Data from four patients over three experiments. **B.** Cells after 14 days of differentiation were evaluated using Zombie Yellow (Biolegend) cell viability dye and utilizing flow cytometry. **B1:** Representative histograms from experiments are shown. **B2:** For each individual experiment, the % viability of patient cells was divided by the percentage measured from normal volunteer (control) cells, and this ratio was plotted. Data from four patients over four experiments. **C.** Cells after 14 days of differentiation were stained with CD66b, CD11b, CD14 and CD15 surface markers and analyzed by flow cytometry. **C1:** Representative histograms from experiments are shown. **C2:** For each individual experiment, the percentage of patient cells expressing a mature phenotype was divided by the percentage measured from normal volunteer (control) cells, and this ratio was plotted. Data from four patients over four experiments. **D**. Cell cytospins stained with Kwik-Diff (eosin/methylene blue) were imaged using a Nikon digital camera. Cell differentiation was evaluated at 400x magnification by light microscope.

The iPSC derived CD34^+^ cells were further pushed towards the myeloid differentiation using the protocol published recently by our group [11,12]. After the 14 days of differentiation there was a sizable drop in cell proliferation in all patient cell lines (Figure 3A). There was also a noticeable cell death in patient cell lines (Figures 3B–1, 3B–2, and 3D). The myeloid differentiation in the patients’ cells, measured by flow cytometry and assessed with surface markers CD66b, CD14, CD11b, CD15, was substantially impaired (Figures 3C–1 and 3C–2).

### CRISPR/Cas9 assisted repair of R736C mutation

**Figure 4.**
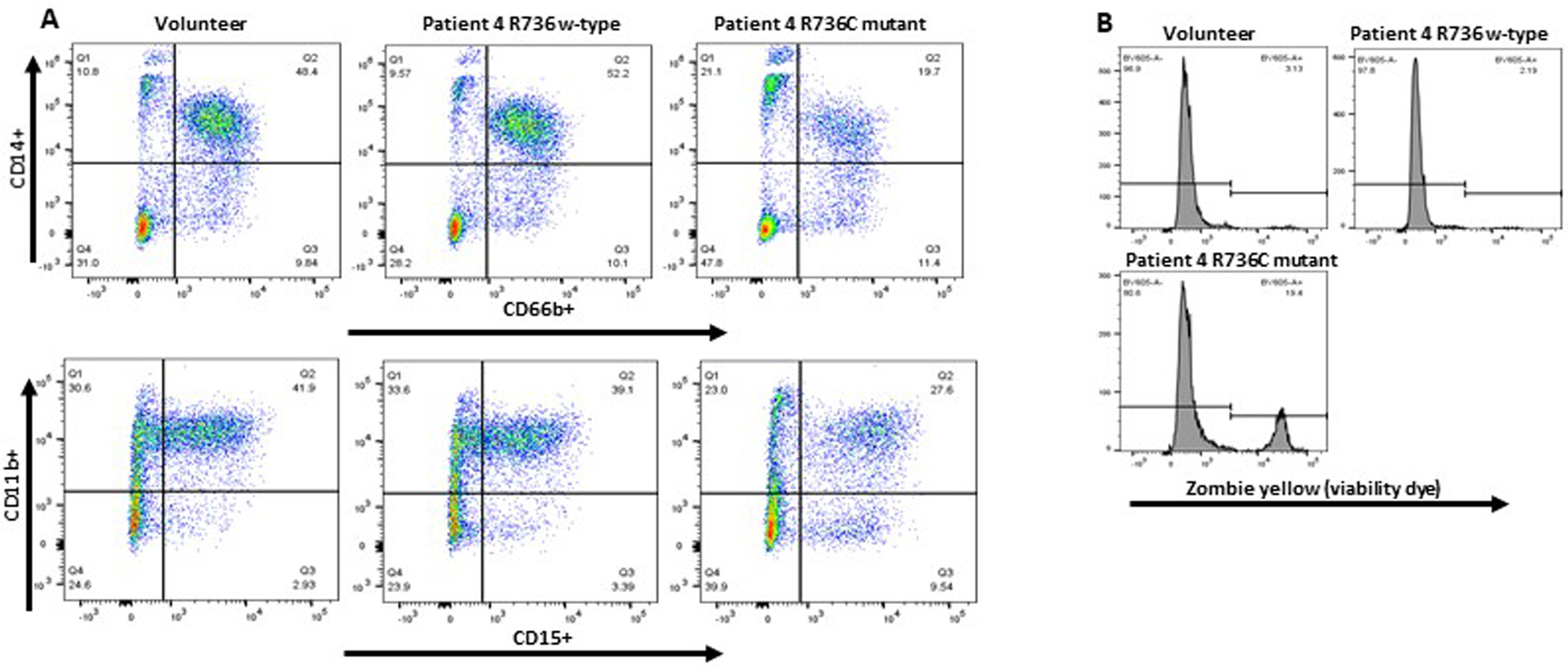
Myeloid differentiation and survival of *TCIRG1* R736C mutation repaired iPSC derived CD34^+^ hematopoietic cells. Patient 4 derived iPSCs with the corrected mutation pushed towards the myeloid differentiation using the standard protocol previously described [11,12]. **A.** Cells after 14 days of differentiation were stained with CD66b, CD14, CD11b, and CD15 surface markers and analyzed by flow cytometry. **B.** Cells after 14 days of differentiation were evaluated using Zombie Yellow (Biolegend) cell viability dye and utilizing flow cytometry.

To validate the causative effect of *TCIRG1* mutations on the maturation, survival, and proliferation of hematopoietic CD34^+^ cells in our cell lines, we conducted a “reverse genetics” experiment. Using CRISPR/Cas9 gene editing, we repaired the R736C mutation in iPSCs derived from Patient 4 (#201 of Family 2). We then performed hematopoietic and subsequent myeloid differentiation experiments with this edited cell line, comparing the results to those from a healthy volunteer and an equally manipulated but unedited R736C-mutant cell line from the same patient. The mutation-repaired cell line exhibited normal differentiation and survival characteristics, similar to the volunteer-derived cell lines (Figure 4).

### TCIRG1 protein intracellular localization

We performed TCIRG1 protein intracellular localization experiments of iPSC differentiated granulocytes utilizing immunostaining and confocal fluorescence microscopy.

**Figure 5.**
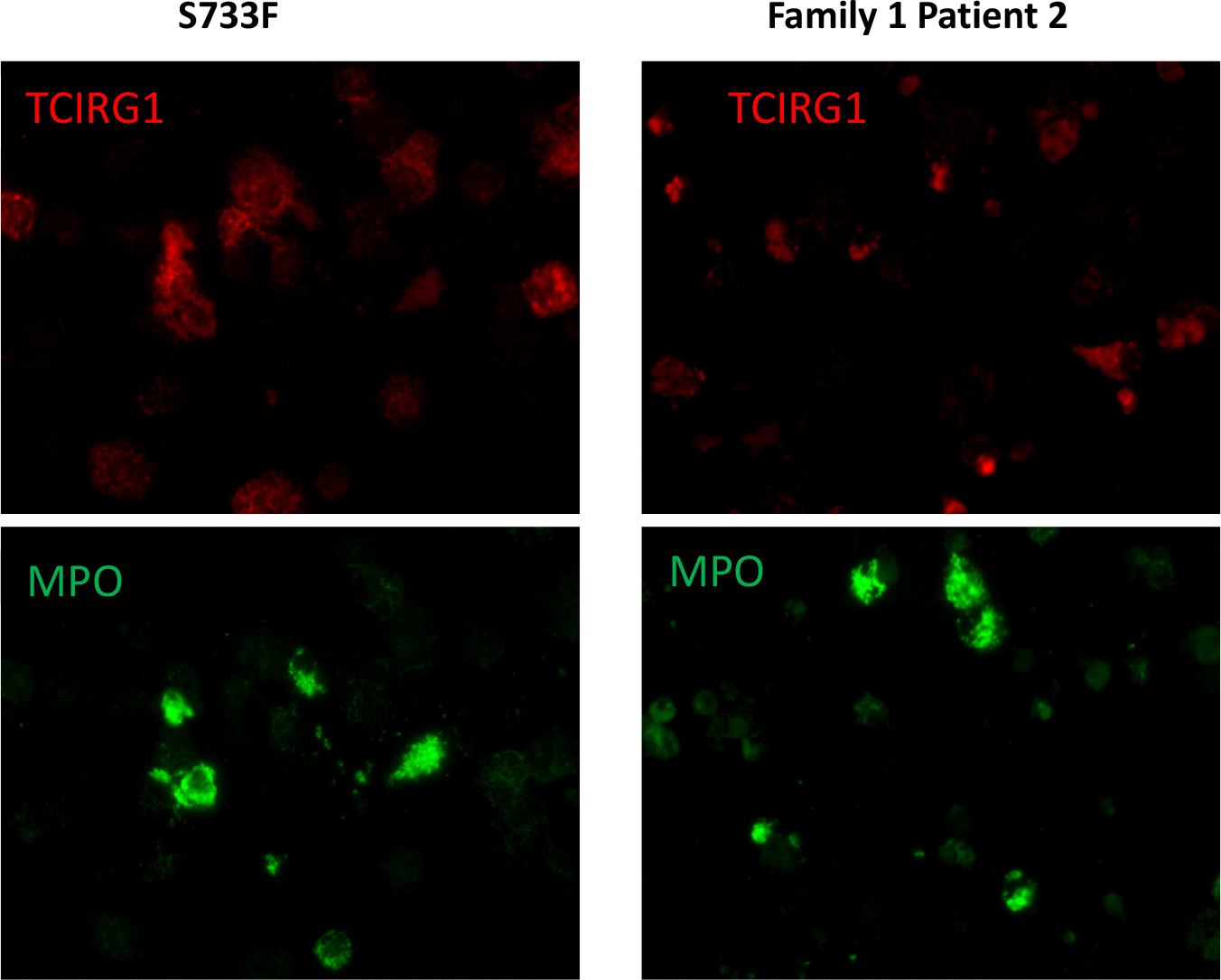
TCIRG1 and MPO immunostaining of iPSC derived granulocytes (representative slides of Patient 2 from Family 1 and S733F CTRL cell line). iPSC derived CD34^+^ cells pushed towards the myeloid differentiation using the standard protocol previously described [11,12]. Cells after 14 days of differentiation were stained with anti-TCIRG1 (Abcam, ab224654) and anti-MPO antibodies (R&D Systems, MAB3174) and evaluated under the immunofluorescence confocal microscope.

In these experiments, we used 4 patient derived cell lines from the families 1 and 2, as well as “benign” S733F cell lines and healthy volunteer. In order to correlate TCIRG1 localization in the cells, we also performed myeloperoxidase (MPO) staining, an abundantly expressed neutrophil protein localized in the primary granules. These studies revealed diffused and unstructured intracellular localization and decreased expression of TCIRG1 protein throughout the cytosol in all 4 mutant cell lines. TCIRG1 staining in both control cell lines (volunteer and S733F) retained granularity, with localization in the subcellular granules. MPO staining was unaltered in all cell lines (Figure 5).

## Discussion

In this study, we utilized patient-derived induced pluripotent stem cells (iPSCs) to model *TCIRG1*-associated neutropenia and elucidate the mechanistic consequences of *TCIRG1* mutations on myeloid differentiation. Our findings provide strong evidence that heterozygous missense mutations at the R736 and E722 positions impair neutrophil development, survival and maturation. Through *in vitro* differentiation studies and CRISPR/Cas9-mediated gene correction, we demonstrated that the observed hematopoietic defects are directly attributable to these mutations, supporting their pathogenic role in congenital neutropenia.

Our data reveal that patient-derived iPSCs with *TCIRG1* mutations exhibit significantly reduced proliferation and increased cell death during myeloid differentiation. These defects were accompanied by a marked reduction in neutrophil-specific markers (CD66b, CD14, CD15, and CD11b) and impaired granulocytic maturation, as observed through flow cytometry and cytospin analyses. Importantly, genome editing to correct the R736C mutation restored normal neutrophil differentiation, confirming that this mutation is causative.

Our immunofluorescence studies revealed an altered intracellular localization of *TCIRG1* in patient-derived cells, suggesting that these mutations disrupt its proper trafficking or retention within cellular compartments. The effect of these mutations on the protein structure is predicted to be “probably damaging” based on Polyphen-2 SNP data. These findings align with prior research demonstrating that mutations in *TCIRG1* can compromise osteoclast activity and, perhaps, an as-yet unidentified mechanism involved in the neutrophil maturation process.

Interestingly, despite the presence of numerous heterozygous *TCIRG1* variants in the general population, only mutations affecting residues R736 and E722 have been associated with neutropenia—this is in contrast to hundreds of other known heterozygous *TCIRG1* mutations that have not been reported to cause this phenotype.

*TCIRG1* is located at 11q13, consists of 20 exons and encodes subunit a3, a 116-kD component (OC116), of V-ATPases. These are vacuolar proton pumps which mediate H^+^ transport through intracellular compartments and organelles of eukaryotic cells, including the phagocytic vacuoles of neutrophils. V-ATPase is composed of two main functional domains: peripheral V1 and membrane associated V0. Both domains consist of multiple subunits; a3 is the largest subunit in V0 domain and has trans-membrane localization. *TCIRG1* is highly expressed in myeloid progenitors, osteoclasts and is essential for bone resorption [6–8].

Our understanding of *TCIRG1* and how it may cause neutropenia is incomplete. Biallelic loss-of-function mutations of *TCIRG1* are the major cause of infantile malignant osteopetrosis (IMO) (OMIM 259700), a lethal autosomal recessive disorder with secondary hematological and neurological abnormalities [9,10,13]. Loss of *TCIRG1* leads to impaired acidification at the ruffle border interface between osteoclasts and bone, which results in impaired bone resorption [14,15]. Osteoclasts regulate osteoblasts, a cell population implicated in hematopoietic stem cell maintenance [16,17] and mobilization of hematopoietic progenitor cells [18]. Studies of RNA profiling of murine osteoblasts indicate that *TCIRG1* is expressed in this cell population [19]. Thus, it is possible that R736S, R736C, R736P and E722D *TCIRIG1* mutations, observed in the neutropenic patients, may indirectly disrupt granulopoiesis by altering the bone marrow microenvironment. Of particular relevance to SCN, RNA expression profiling of granulocytic precursors from healthy donors suggests that *TCIRG1* is highly expressed in promyelocytes (Human Protein Atlas). Thus, these variants may also directly act on granulocytic precursors to impair their differentiation.

Other studies have shown that *TCIRG1* through alternative splicing and usage of an alternative initiation codon in exon 7 gives rise to the membrane protein TIRC7 demonstrated to play an essential role for the immune response [20]. There is evidence that more than two transcript variants of *TCIRG1* are expressed in human tissues (e.g., heart, liver, kidney, lung, pancreas) [21]. Studies in mice have shown that homozygous mutation at R740S (analogous to human R736 position) is lethal in mice and heterozygous mutation of the V-ATPase a3 subunit R740S causes dominant negative osteopetrosis [14]. RNA interference to silence TIRC7, Ap6i +/− mice, reduced inflammatory responses in a mouse model of periodontal disease and decreased osteoclasts and monocytes [22]. These reports do not comment on granulocytopoiesis. Studies in yeast have shown that missense mutations in R735 (analog for the human R736 position) disrupt the functional integrity of vacuolar V-ATPase and thereby disrupt proton pumping, a selective effect of this specific mutation [23,24]. These studies infer that these missense mutations at R736 may act in a dominant fashion. Neutropenia with mutations R736S, R736C, and R736P occur at this exact location. Although direct structural data on *TCIRG1* is lacking, arginine at position 736 and glutamic acid at position722 lie outside of the ATPase’s V0 domain which is responsible for proton translocation and are evolutionarily highly conserved [24]. We further investigated the roles of R736 and E722 by examining high-resolution cryo-EM structures of a homologous yeast V-ATPase (PDB: 6C6L, 6M0R) [25,26]. These structural data, along with accompanying molecular dynamics simulations, demonstrate that the guanidino group of R736 and the carboxylic acid group of E722 are key components of the proton transport pathway that underlies ATP hydrolysis–driven proton pumping by the V-ATPase. Substituting R736 with residues such as cysteine, proline, or serine—which lack guanidino groups—or replacing E722 with an aspartate residue, would be expected to disrupt proton transport and thereby impair V-ATPase activity in neutrophils. Indeed, even a conservative substitution of lysine at the equivalent R736 position in yeast has been shown to abolish function [24]. Although the structural consequences of these mutations are well understood, the connection between impaired proton transport and the clinical phenotype of congenital neutropenia observed in patients heterozygous for R736 or E722 mutations remains to be fully elucidated. Finally, mutations at S733 are not predicted to interfere with proton transport based on the proposed mechanism [26], consistent with the benign nature of the S733F variant in the general population and supporting its use as a negative control in this study.

### Tissue-specific effects of *TCIRG1* mutations

One of the most intriguing aspects of *TCIRG1*-associated neutropenia is the apparent lack of defects in other tissues, despite the gene’s well-established role in osteoclast function and expression in other tissues. This suggests that neutrophils may have a unique dependence on *TCIRG1*-mediated V-ATPase activity, possibly due to their reliance on vesicular trafficking for granule formation and intracellular signaling. Recent publications continue to reveal the increasingly multifaceted roles of neutrophils, including their involvement in tumor microenvironment homeostasis, wound healing, and other processes beyond their traditional function in host defense [27–29]. Unlike osteoclasts, which exhibit redundancy in acidification mechanisms [30], neutrophil precursors may be uniquely vulnerable to disruptions in *TCIRG1* function, leading to impaired differentiation and survival.

Another possibility is that alternative compensatory pathways exist in non-hematopoietic tissues, allowing them to circumvent the effects of *TCIRG1* mutations. In neutrophils, however, these compensatory mechanisms may be absent or insufficient to maintain proper V-ATPase function, leading to selective lineage defects. It is well-known that during maturation, neutrophils lose some key organelles, like mitochondria, Golgi apparatus and have very few ribosomes [31,32]. Further research into the tissue-specific expression patterns and regulatory networks governing *TCIRG1* function will be crucial in unraveling these mechanisms.

### Hypothesis: Gain-of-function effects in TCIRG1 neutropenia

An alternative hypothesis is that the molecular mechanism of *TCIRG1*-associated neutropenia is not due to a simple loss-of-function, but rather a gain-of-function effect caused by the specific mutations at R736 and E722. These mutations may induce aberrant intracellular trafficking of *TCIRG1*, leading to its mis-localization and subsequent disruption of normal vesicular pathways. Our experiments on the intracellular localization of the *TCIRG1* protein support this hypothesis. This could trigger an unfolded protein response (UPR) and promote apoptosis in neutrophil progenitors, selectively impairing their differentiation and survival. Given that neutrophils have a highly specialized and dynamic vesicular system required for granule formation and antimicrobial function, even minor disruptions in trafficking may have profound effects on their development. Further studies are needed to explore whether these mutations interfere with protein degradation pathways, vesicle fusion, or cellular stress responses in neutrophil precursors.

Similar gain-of-function effects causing UPR and apoptosis have been described in other autosomal dominant genetic diseases. For instance, mutations in *ELANE*, which encodes neutrophil elastase, lead to severe congenital neutropenia (SCN) through the accumulation of misfolded protein and ER stress, resulting in increased cell death of neutrophil precursors [33,34]. Similarly, autosomal dominant mutations in SOD1 associated with amyotrophic lateral sclerosis (ALS) induce toxic protein aggregation, causing oxidative stress and neuronal apoptosis [35,36]. Another example is hereditary transthyretin amyloidosis, where misfolded transthyretin proteins aggregate and cause cytotoxicity in peripheral nerves and heart [37]. These parallels suggest that the heterozygous mutations in *TCIRG1* at R736 and E722 may also induce a pathogenic gain-of-function effect through disruption of protein homeostasis, leading to neutrophil-specific cellular stress and apoptosis.

### Clinical implications and future directions

The identification of *TCIRG1* mutations as a genetic cause of congenital neutropenia has important clinical implications for diagnosis and patient management. The severity of neutropenia and associated risk of infectious complications varies within the six families, suggesting that, future studies should investigate potential genetic modifiers that may influence disease severity. Additionally, therapeutic strategies aimed at restoring V-ATPase function, either through targeted small molecules or gene correction approaches, warrant further exploration.

As the unfolded protein response and apoptosis appear to play a central role in *TCIRG1*-associated neutropenia, experimental therapeutic approaches targeting these pathways should be explored. Apoptosis inhibitors such as caspase inhibitors (e.g., Emricasan) or BH3 mimetics (e.g., Venetoclax) may provide protection against excessive cell death in neutrophil progenitors [38,39]. Additionally, pharmacological UPR modulators such as tauroursodeoxycholic acid (TUDCA) or salubrinal, which help alleviate ER stress, could be investigated as potential interventions to rescue neutrophil differentiation and survival [40–43]. Future studies should evaluate the efficacy of these compounds in patient-derived cell models to determine their potential therapeutic benefit.

Our study also raises intriguing questions regarding the broader role of *TCIRG1* in hematopoiesis. While traditionally associated with osteoclast function, our data suggests that some *TCIRG1* mutations are critical for neutrophil survival. Further research is needed to define the specific cellular pathways disrupted by these *TCIRG1* mutations and determine whether these findings extend to other hematopoietic lineages.

## Conclusion

In summary, our study provides compelling evidence that some heterozygous *TCIRG1* mutations disrupt myeloid differentiation, survival and maturation of neutrophils. By leveraging patient-derived iPSCs and genome editing, we have established a mechanistic link between *TCIRG1* dysfunction and congenital neutropenia. These findings not only enhance our understanding of neutrophil development but also pave the way for potential therapeutic interventions in *TCIRG1*-associated hematological disorders.

## Supplementary Material

JCI-25-228-Supplementary-File

## Figures and Tables

**Table 1.** Clinical and genetic characteristics of patients from 6 families with *TCIRG1* mutations.

Family #	Family ANC Range (×10^9^/L)	Family Clinical Problems	*TCIRG1* Mutation
1	0.054 – 2.232	Mouth ulcers, skin infections, oral abscesses, perirectal abscesses, liver abscesses	R736S
2	0.056 – 8.500	Gingivitis, mouth ulcers, otitis media, tonsillitis, pneumonia	R736C
3	0.160 – 0.900	Mouth ulcers, oral abscess, bronchitis, sinusitis, otitis media, pneumonia, sepsis (hospitalized)	R736C
4	0.043 – 5.400	Growth failure, skin infections, gingivitis, multiple febrile episodes requiring hospitalization, otitis media, rectal ulcer, tuberculosis	E722D
5	0.200 – 1.000	Mouth ulcers, recurrent urinary tract infection, skin infection, mastitis complicated by abscess	R736C
6	0.100 – 1.600	Pneumonia resulting in hospitalization	R736P

The table summarizes the absolute neutrophil count (ANC) ranges, observed clinical problems, and the identified *TCIRG1* mutations for each family. The clinical problems observed in each family are described above and are consistent with the typical phenotype associated with congenital neutropenia.

## References

[1] Skokowa J, Dale DC, Touw IP, Zeidler C, Welte K. Severe congenital neutropenias. Nat Rev Dis Primers. 2017 Jun 8;3:17032. 28593997 10.1038/nrdp.2017.32 PMC5821468

[2] Dale DC, Guerry D 4th, Wewerka JR, Bull JM, Chusid MJ. Chronic neutropenia. Medicine (Baltimore). 1979 Mar;58(2):128–44. 431399 10.1097/00005792-197903000-00002

[3] Makaryan V, Rosenthal EA, Bolyard AA, Kelley ML, Below JE, Bamshad MJ, TCIRG1-associated congenital neutropenia. Hum Mutat. 2014 Jul;35(7):824–7. 24753205 10.1002/humu.22563 PMC4055522

[4] Rosenthal EA, Makaryan V, Burt AA, Crosslin DR, Kim DS, Smith JD, Association Between Absolute Neutrophil Count and Variation at TCIRG1: The NHLBI Exome Sequencing Project. Genet Epidemiol. 2016 Sep;40(6):470–4. 27229898 10.1002/gepi.21976 PMC5079157

[5] Shinwari K, Rehman HM, Liu G, Bolkov MA, Tuzankina IA, Chereshnev VA. Novel Disease-Associated Missense Single-Nucleotide Polymorphisms Variants Predication by Algorithms Tools and Molecular Dynamics Simulation of Human TCIRG1 Gene Causing Congenital Neutropenia and Osteopetrosis. Front Mol Biosci. 2022 Apr 28;9:879875. 35573728 10.3389/fmolb.2022.879875 PMC9095858

[6] Hinton A, Bond S, Forgac M. V-ATPase functions in normal and disease processes. Pflugers Arch. 2009 Jan;457(3):589–98. 18026982 10.1007/s00424-007-0382-4

[7] Frattini A, Orchard PJ, Sobacchi C, Giliani S, Abinun M, Mattsson JP, Defects in TCIRG1 subunit of the vacuolar proton pump are responsible for a subset of human autosomal recessive osteopetrosis. Nat Genet. 2000 Jul;25(3):343–6. 10888887 10.1038/77131

[8] Forgac M Vacuolar ATPases: rotary proton pumps in physiology and pathophysiology. Nat Rev Mol Cell Biol. 2007 Nov;8(11):917–29. 17912264 10.1038/nrm2272

[9] Susani L, Pangrazio A, Sobacchi C, Taranta A, Mortier G, Savarirayan R, TCIRG1-dependent recessive osteopetrosis: mutation analysis, functional identification of the splicing defects, and in vitro rescue by U1 snRNA. Hum Mutat. 2004 Sep;24(3):225–35. 15300850 10.1002/humu.20076

[10] Villa A, Guerrini MM, Cassani B, Pangrazio A, Sobacchi C. Infantile malignant, autosomal recessive osteopetrosis: the rich and the poor. Calcif Tissue Int. 2009 Jan;84(1):1–12. 19082854 10.1007/s00223-008-9196-4

[11] Sabo P, Makaryan V, Dicken Y, Povodovski L, Rockah L, Bar T, Mutant allele knockout with novel CRISPR nuclease promotes myelopoiesis in ELANE neutropenia. Mol Ther Methods Clin Dev. 2022 Jun 9;26:119–31. 35795780 10.1016/j.omtm.2022.06.002 PMC9240714

[12] Makaryan V, Kelley M, Bolyard AA, Chugh G, Dale DC. Evaluation of Neutrophil Elastase Inhibitors as Potential Therapies for ELANE Associated Neutropenia. J Cell Immunol. 2024;6(5):211–8. 39867870 10.33696/immunology.6.208 PMC11759478

[13] Sobacchi C, Frattini A, Orchard P, Porras O, Tezcan I, Andolina M, The mutational spectrum of human malignant autosomal recessive osteopetrosis. Hum Mol Genet. 2001 Aug 15;10(17):1767–73. 11532986 10.1093/hmg/10.17.1767

[14] Ochotny N, Flenniken AM, Owen C, Voronov I, Zirngibl RA, Osborne LR, Henderson JE, Adamson SL, Rossant J, Manolson MF, Aubin JE. The V-ATPase a3 subunit mutation R740S is dominant negative and results in osteopetrosis in mice. J Bone Miner Res. 2011 Jul;26(7):1484–93. 21305608 10.1002/jbmr.355

[15] Kawamura N, Tabata H, Sun-Wada GH, Wada Y. Optic nerve compression and retinal degeneration in Tcirg1 mutant mice lacking the vacuolar-type H-ATPase a3 subunit. PLoS One. 2010 Aug 10;5(8):e12086. 20711468 10.1371/journal.pone.0012086 PMC2919411

[16] Calvi LM, Adams GB, Weibrecht KW, Weber JM, Olson DP, Knight MC, Osteoblastic cells regulate the haematopoietic stem cell niche. Nature. 2003 Oct 23;425(6960):841–6. 14574413 10.1038/nature02040

[17] Zhang M, Xuan S, Bouxsein ML, von Stechow D, Akeno N, Faugere MC, Osteoblast-specific knockout of the insulin-like growth factor (IGF) receptor gene reveals an essential role of IGF signaling in bone matrix mineralization. J Biol Chem. 2002 Nov 15;277(46):44005–12. 12215457 10.1074/jbc.M208265200

[18] Kollet O, Dar A, Shivtiel S, Kalinkovich A, Lapid K, Sztainberg Y, Osteoclasts degrade endosteal components and promote mobilization of hematopoietic progenitor cells. Nat Med. 2006 Jun;12(6):657–64. 16715089 10.1038/nm1417

[19] Eash KJ, Greenbaum AM, Gopalan PK, Link DC. CXCR2 and CXCR4 antagonistically regulate neutrophil trafficking from murine bone marrow. J Clin Invest. 2010 Jul;120(7):2423–31. 20516641 10.1172/JCI41649 PMC2898597

[20] Heinemann T, Bulwin GC, Randall J, Schnieders B, Sandhoff K, Volk HD, Genomic organization of the gene coding for TIRC7, a novel membrane protein essential for T cell activation. Genomics. 1999 May 1;57(3):398–406. 10329006 10.1006/geno.1999.5751

[21] Smirnova AS, Morgun A, Shulzhenko N, Silva ID, Gerbase-DeLima M. Identification of new alternative splice events in the TCIRG1 gene in different human tissues. Biochem Biophys Res Commun. 2005 May 13;330(3):943–9. 15809087 10.1016/j.bbrc.2005.03.065

[22] Jiang H, Chen W, Zhu G, Zhang L, Tucker B, Hao L, RNAi-mediated silencing of Atp6i and Atp6i haploinsufficiency prevents both bone loss and inflammation in a mouse model of periodontal disease. PLoS One. 2013;8(4):e58599. 23577057 10.1371/journal.pone.0058599 PMC3618217

[23] Coonrod EM, Graham LA, Carpp LN, Carr TM, Stirrat L, Bowers K, Homotypic vacuole fusion in yeast requires organelle acidification and not the V-ATPase membrane domain. Dev Cell. 2013 Nov 25;27(4):462–8. 24286827 10.1016/j.devcel.2013.10.014 PMC4086684

[24] Kawasaki-Nishi S, Nishi T, Forgac M. Arg-735 of the 100-kDa subunit a of the yeast V-ATPase is essential for proton translocation. Proc Natl Acad Sci U S A. 2001 Oct 23;98(22):12397–402. 11592980 10.1073/pnas.221291798 PMC60065

[25] Roh SH, Stam NJ, Hryc CF, Couoh-Cardel S, Pintilie G, Chiu W, The 3.5-Å CryoEM Structure of Nanodisc-Reconstituted Yeast Vacuolar ATPase Vo Proton Channel. Mol Cell. 2018 Mar 15;69(6):993–1004.e3. 29526695 10.1016/j.molcel.2018.02.006 PMC5893162

[26] Roh SH, Shekhar M, Pintilie G, Chipot C, Wilkens S, Singharoy A, Cryo-EM and MD infer water-mediated proton transport and autoinhibition mechanisms of Vo complex. Sci Adv. 2020 Oct 7;6(41):eabb9605. 33028525 10.1126/sciadv.abb9605 PMC7541076

[27] Awasthi D, Sarode A. Neutrophils at the Crossroads: Unraveling the Multifaceted Role in the Tumor Microenvironment. Int J Mol Sci. 2024 Mar 2;25(5):2929. 38474175 10.3390/ijms25052929 PMC10932322

[28] Zhang M, Qin H, Wu Y, Gao Q. Complex role of neutrophils in the tumor microenvironment: an avenue for novel immunotherapies. Cancer Biol Med. 2024 Sep 19;21(10):849–63. 39297568 10.20892/j.issn.2095-3941.2024.0192 PMC11523270

[29] Renò F, Pagano CA, Bignotto M, Sabbatini M. Neutrophil Heterogeneity in Wound Healing. Biomedicines. 2025 Mar 12;13(3):694. 40149670 10.3390/biomedicines13030694 PMC11940162

[30] Barvencik F, Kurth I, Koehne T, Stauber T, Zustin J, Tsiakas K, CLCN7 and TCIRG1 mutations differentially affect bone matrix mineralization in osteopetrotic individuals. J Bone Miner Res. 2014 Apr;29(4):982–91. 24108692 10.1002/jbmr.2100

[31] Peng S, Gao J, Stojkov D, Yousefi S, Simon HU. Established and emerging roles for mitochondria in neutrophils. Immunol Rev. 2023 Mar;314(1):413–26. 36331270 10.1111/imr.13158

[32] Rožman S, Yousefi S, Oberson K, Kaufmann T, Benarafa C, Simon HU. The generation of neutrophils in the bone marrow is controlled by autophagy. Cell Death Differ. 2015 Mar;22(3):445–56. 25323583 10.1038/cdd.2014.169 PMC4326574

[33] Nayak RC, Trump LR, Aronow BJ, Myers K, Mehta P, Kalfa T, Pathogenesis of ELANE-mutant severe neutropenia revealed by induced pluripotent stem cells. J Clin Invest. 2015 Aug 3;125(8):3103–16. 26193632 10.1172/JCI80924 PMC4563755

[34] Nanua S, Murakami M, Xia J, Grenda DS, Woloszynek J, Strand M, Activation of the unfolded protein response is associated with impaired granulopoiesis in transgenic mice expressing mutant Elane. Blood. 2011 Mar 31;117(13):3539–47. 21285438 10.1182/blood-2010-10-311704 PMC3072877

[35] Pasinelli P, Belford ME, Lennon N, Bacskai BJ, Hyman BT, Trotti D, Amyotrophic lateral sclerosis-associated SOD1 mutant proteins bind and aggregate with Bcl-2 in spinal cord mitochondria. Neuron. 2004 Jul 8;43(1):19–30. 15233914 10.1016/j.neuron.2004.06.021

[36] Vehviläinen P, Koistinaho J, Gundars G. Mechanisms of mutant SOD1 induced mitochondrial toxicity in amyotrophic lateral sclerosis. Front Cell Neurosci. 2014 May 9;8:126. 24847211 10.3389/fncel.2014.00126 PMC4023018

[37] Ton VK, Mukherjee M, Judge DP. Transthyretin cardiac amyloidosis: pathogenesis, treatments, and emerging role in heart failure with preserved ejection fraction. Clin Med Insights Cardiol. 2015 Jan 5;8(Suppl 1):39–44. 25628512 10.4137/CMC.S15719 PMC4284988

[38] Nassauer L, Schott JW, Harre J, Warnecke A, Morgan M, Galla M, The caspase-inhibitor Emricasan efficiently counteracts cisplatin- and neomycin-induced cytotoxicity in cochlear cells. J Mol Med (Berl). 2024 Sep;102(9):1163–74. 39110182 10.1007/s00109-024-02472-2 PMC11358181

[39] Lin VS, Xu ZF, Huang DCS, Thijssen R. BH3 Mimetics for the Treatment of B-Cell Malignancies-Insights and Lessons from the Clinic. Cancers (Basel). 2020 Nov 12;12(11):3353. 33198338 10.3390/cancers12113353 PMC7696913

[40] Kusaczuk M Tauroursodeoxycholate-Bile Acid with Chaperoning Activity: Molecular and Cellular Effects and Therapeutic Perspectives. Cells. 2019 Nov 20;8(12):1471. 31757001 10.3390/cells8121471 PMC6952947

[41] Song H, Liu J, Wang L, Hu X, Li J, Zhu L, Tauroursodeoxycholic acid: a bile acid that may be used for the prevention and treatment of Alzheimer’s disease. Front Neurosci. 2024 Feb 19;18:1348844. 38440398 10.3389/fnins.2024.1348844 PMC10909943

[42] Reijonen S, Putkonen N, Nørremølle A, Lindholm D, Korhonen L. Inhibition of endoplasmic reticulum stress counteracts neuronal cell death and protein aggregation caused by N-terminal mutant huntingtin proteins. Exp Cell Res. 2008 Mar 10;314(5):950–60. 18255062 10.1016/j.yexcr.2007.12.025

[43] Wang ZF, Gao C, Chen W, Gao Y, Wang HC, Meng Y, Salubrinal offers neuroprotection through suppressing endoplasmic reticulum stress, autophagy and apoptosis in a mouse traumatic brain injury model. Neurobiol Learn Mem. 2019 May;161:12–25. 30851432 10.1016/j.nlm.2019.03.002

