# Supplementary material for: Modeling TCIRG1 Neutropenia by Utilizing Patient Derived Induced Pluripotent Stem Cells
Source: J Cell Immunol. Author manuscript; Available in PMC 2025 Sep 17. (PMC12439587; doi:10.33696/immunology.7.228)
Supplement: JCI-25-228-Supplementary-File [file NIHMS2101044-supplement-JCI-25-228-Supplementary-File.pdf]

Makaryan V, Kelley ML, Bolyard AA, Cavanaugh C, Hesson J, Mathieu J, et al. Modeling TCIRG1 Neutropenia by Utilizing Patient Derived Induced Pluripotent Stem Cells. J Cell Immunol. 2025;7(3):98-112.

Supplementary Figure 1.

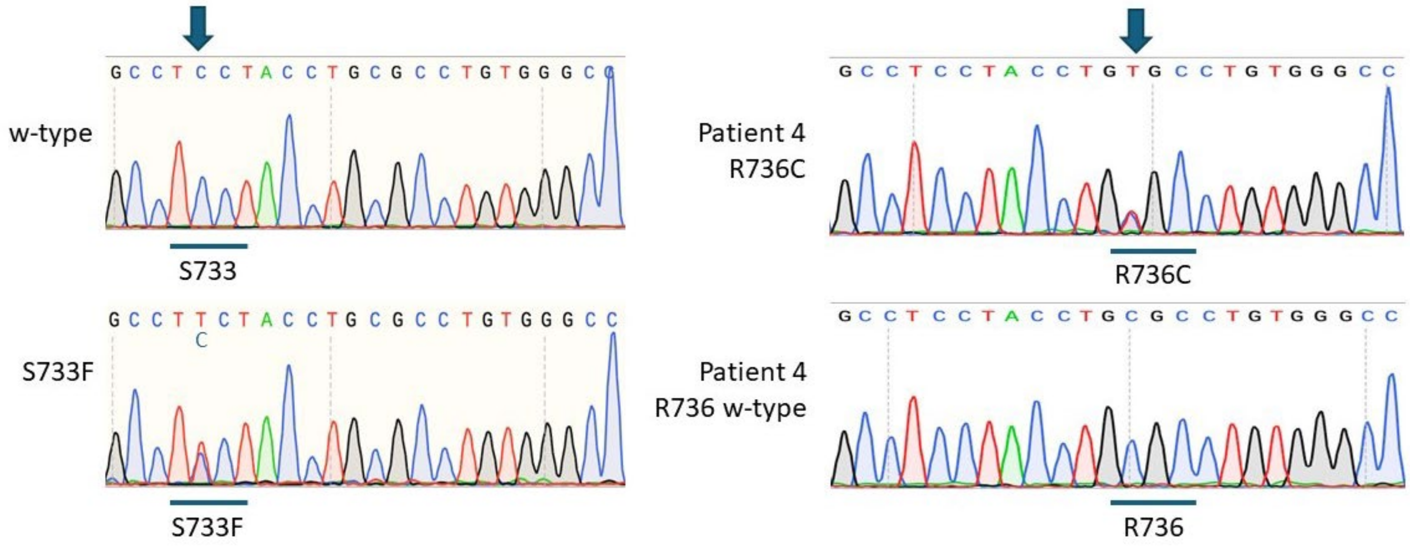

Supplementary Figure 1. Electropherograms of iPSC lines before and after CRISPR/Cas9 editing. *Left:* Introduction of the *TCIRG1* S733F mutation into a healthy volunteer-derived iPSC line. *Right:* Correction of the *TCIRG1* R736C mutation in Patient 4-derived iPSCs.
